# Supplementary material for: Profiling 25 Bone Marrow microRNAs in Acute Leukemias and Secondary Nonleukemic Hematopoietic Conditions
Source: Biomedicines. 2020 Dec 14;8(12):607. doi: 10.3390/biomedicines8120607 (PMC7764834; doi:10.3390/biomedicines8120607)
Supplement: Supplementary file 1 [file biomedicines-08-00607-s001.zip › biomedicines-1009119-supplementary/Supplementary Kovynev/Supplementary. Table 1..docx]

Table 1. Patient characteristics at the time of diagnosis.

| **Clinical data of acute lymphoblastic leukemia (ALL) patients (n=22).** | | | | |
| --- | --- | --- | --- | --- |
|  |  |  |  |  |
| **Characteristic** | **N (%)** |  |  |  |
| Gender |  |  |  |  |
| Male | 10(45) |  |  |  |
| Female | 12(55) |  |  |  |
| Age |  |  |  |  |
| < 60 years | 17(77) |  |  |  |
| ≥60 years | 5(23) |  |  |  |
| Median WBC count, × 10^9^/L | 26,7 |  |  |  |
| Median BM blast, % | 25,6 |  |  |  |
| Cytogenetic test |  |  |  |  |
| Ph chromosome(+) | 2(9) |  |  |  |
| Ph chromosome(–) | 20(91) |  |  |  |
| Immunophenotype |  |  |  |  |
| Early Pre-B ALL | 3(14) |  |  |  |
| Common ALL | 11(50) |  |  |  |
| Pre-B ALL | 2(9) |  |  |  |
| Mature B-cell ALL | 1(5) |  |  |  |
| Pre-T ALL | 1(5) |  |  |  |
| Pro-T ALL | 2(9) |  |  |  |
| Cortical T | 2(9) |  |  |  |
|  |  |  |  |  |
| Abbreviations: WBC = white blood cell; BM = bone marrow. | | | | |

| **Clinical characteristics of acute myeloblastic leukemia (AML) patients at diagnosis (n=44).** | |
| --- | --- |
|  |  |
| **Characteristic** | **N (%)** |
| Gender |  |
| Male | 16(36) |
| Female | 28(64) |
| Age |  |
| < 60 years | 29(66) |
| ≥60 years | 15(34) |
| Median WBC count, × 10^9^/L | 29,5 |
| Median BM blast, % | 31,7 |
| FAB |  |
| M0 | 4(9) |
| M1 | 7(16) |
| M2 | 11(25) |
| M3 | 12(27) |
| M4 | 5(11) |
| M5 | 5(11) |
| Cytogenetic test |  |
| PML/RARA (t(15;17)(q24;q21)) | 7(16) |
| Trisomy 8 | 1(2) |
| Trisomy 11 | 1(2) |
| MLL (11q23.3) | 3(7) |
| RUNX1/RUNX1T1 t(8;21)(q22(;q22)) | 3(7) |

Abbreviations: WBC = white blood cell; BM = bone marrow; FAB = French-American-British.
